# Wissenserwerb, Wissensstand und Wissenstransfer in der Kompressionstherapie: Querschnittsstudie in Gesundheitsberufen, die phlebologische Kompressionsversorgungen anwenden

**DOI:** 10.1007/s00105-024-05314-x

**Published:** 2024-02-27

**Authors:** Kerstin Protz, Joachim Dissemond, Matthias Augustin, Toni Maria Janke

**Affiliations:** 1 CWC – Comprehensive Wound Center, Institute for Health Services Research in Dermatology and Nursing (IVDP), University Medical Center Hamburg-Eppendorf (UKE), Martinistr. 52, 20246 Hamburg, Deutschland; 2 Department of Dermatology, Venereology and Allergology, University Hospital Essen, Essen, Deutschland

**Keywords:** Angehörige medizinischer und pflegerischer Berufe, Fortbildung, Wissensdefizit, Leitlinien, Expertenstandards, Health care professionals, Continuing medical education, Knowledge deficit, Guidelines, Expert standards

## Abstract

**Hintergrund:**

Im Zuge des wissenschaftlichen Fortschritts sollte bei Mitarbeitern in Gesundheitsberufen regelmäßig eine entsprechende Fortbildung erfolgen. Hierfür ist ein Wissenstransfer essenziell. In dieser Querschnittsstudie sollte daher der Status von Wissenserwerb, Wissensstand und Wissenstransfer der Berufsgruppen, die phlebologische Kompressionsverbände anwenden, in Deutschland untersucht werden.

**Material und Methoden:**

Mitarbeiter in Gesundheitsberufen (Ärzte, Pflegefachkräfte und medizinische Fachangestellte) erhielten einen für diese Studie entwickelten Fragebogen, der verschiedene Aspekte von Erwerb, Stand und Transfer des Wissens abfragte.

**Ergebnisse:**

Die Antworten von 522 Teilnehmern wurden ausgewertet. Das Thema Kompressionstherapie wurde in der Ausbildung bzw. Studium zu 43,3 % nicht unterrichtet. Fachzeitschriften, die Kompressionstherapie thematisieren, wurden von 16,1 % der Teilnehmer regelmäßig (mindestens 6‑mal/Jahr) gelesen, 63,0 % hatten keine Fachbücher zum Thema. Lediglich 6,7 % kannten themenbezogene AWMF(Arbeitsgemeinschaft der Wissenschaftlichen Medizinischen Fachgesellschaften e. V.)-Leitlinien und 16,3 % den entsprechenden DNQP(Deutsches Netzwerk für Qualitätsentwicklung in der Pflege)-Expertenstandard. An mindestens einer internen Fortbildung pro Jahr zur Kompressionstherapie nahmen 41,2 % teil, bei externen Fortbildungen waren es 72,0 % und bei Online-Fortbildungen 19,2 %. Insgesamt gaben 30,7 % an, keine Informationsquellen zum Wissenserwerb zu nutzen.

**Schlussfolgerungen:**

Mögliche Wissensquellen zur Kompressionstherapie in Deutschland sind innerhalb der hier untersuchten Berufsgruppen unzureichend bekannt oder werden nicht regelhaft genutzt. Die Folge daraus ist ein erhebliches Wissensdefizit mit Diskrepanz zwischen dem aktuellen Stand der Wissenschaft und der Praxis.

## Abstract (en)

**Background:**

Due to scientific progress, healthcare professionals should regularly undergo appropriate continuing education. For this, knowledge transfer is essential. Therefore, the aim of this cross-sectional study was to investigate the acquisition, status and transfer of knowledge of professional groups applying phlebological compression therapy in Germany.

**Materials and methods:**

Healthcare professionals (physicians, nurses and medical assistants) received a questionnaire developed for this study, which queried different aspects of acquisition, status and transfer of knowledge.

**Results:**

Responses from 522 participants were analysed. The topic of compression therapy was not taught in the nursing or medical education of 43.3%. Specialist journals that address compression therapy were read regularly (at least 6 times/year) by 16.1% of the participants; 63.0% had no specialist books on this subject. Only 6.7% were aware of AWMF (“Arbeitsgemeinschaft der Wissenschaftlichen Medizinischen Fachgesellschaften”) guidelines on the topic and 16.3% of the corresponding DNQP (“Deutsches Netzwerk für Qualitätsentwicklung in der Pflege”) expert standard. In all, 41.2% participated in at least one internal training on compression therapy per year, 72.0% in external training and 19.2% in online training. A total of 30.7% stated that they did not use any information sources to acquire knowledge.

**Conclusions:**

Possible sources of knowledge about compression therapy in Germany are insufficiently known within the investigated healthcare professional groups studied or are not regularly used. The result is a considerable knowledge deficit with a discrepancy between the current state of science and practice.

Der Wissensstand in Medizin und Pflege entwickelt sich beständig weiter, und Angehörige medizinischer und pflegerischer Berufe sind somit aufgerufen, ihren Wissensstand anzupassen, bestehende Kenntnisse zu prüfen und sich neue Sachverhalte anzueignen. Diese Studie zielte daher darauf ab, Erkenntnisse darüber zu gewinnen, ob und in welcher Weise sich medizinische und pflegerische Berufsgruppen dieser Herausforderung stellen. Beispielhaft wurde die Kompressionstherapie gewählt, da sie Bestandteil beider Versorgungsbereiche ist und im interdisziplinären und multiprofessionellen Umfeld stattfindet.

Erfolgreicher Wissenstransfer ermöglicht, ein erlerntes Konzept zur Lösung eines Problems zu verwenden, das in einem anderen Zusammenhang auftritt [1]. Wissenstransfer findet sowohl innerhalb einer Gruppe statt als auch zwischen Angehörigen verschiedener Gruppen und erfolgt idealerweise angepasst an die individuellen Bedürfnisse und Fähigkeiten des Empfängers [2]. Hinzu kommen weitere Faktoren, die eine unterschiedlich große Rolle spielen können: soziale, kulturelle und ökonomische Aspekte, der Prozess der Wissensvermittlung und deren Kontext, sowie die dabei weitergegebenen Inhalte [3]. Insbesondere bei Mitarbeitern von Gesundheitsberufen gibt es durch wissenschaftlichen Fortschritt eine ständige Weiterentwicklung des vorhandenen Wissens, das bedeutet, die Notwendigkeit zum Wissenserwerb nimmt zu, und der Wissensstand steigt an. Wissenstransfer in diesen Berufsfeldern verläuft zum einen aus dem wissenschaftlichen Diskurs heraus in den Versorgungsalltag hinein [1]. Zum anderen ist die Praxis geprägt durch den Austausch verschiedener Fachdisziplinen untereinander. Diese beiden Aspekte des Wissenstransfers prägen insbesondere den Themenkomplex der Kompressionstherapie von Menschen mit phlebologischen Krankheitsbildern. Im Versorgungsalltag setzen sich wissenschaftliche Erkenntnisse nur langsam durch [4]. Insbesondere in der Kompressionstherapie wird seit Jahren beobachtet, dass die Praxis aktuelle wissenschaftliche Erkenntnisse nicht ausreichend miteinbezieht [5–7]. Voraussetzung für die Übernahme neuer wissenschaftlicher Erkenntnisse in den Versorgungsalltag ist beispielsweise der klare Praxisbezug [1]. In der phlebologischen Kompressionstherapie handelt es sich meist um längere Behandlungsverläufe, die sich sowohl auf den ambulanten als auch den stationären Bereich ausdehnen können [5, 6]. Hierbei hat es der Patient mit verschiedenen Professionen und innerhalb der Professionen mit mehreren Fachdisziplinen zu tun. Entscheidend für den Behandlungserfolg und somit für die Lebensqualität der Betroffenen sind der Informations- und Kenntnisstand der Versorger, ihr koordiniertes Vorgehen und die zeitnahe Anpassung des Therapieplans bei Veränderungen. Die Basis hierfür bildet der Wissenstransfer zwischen allen Akteuren.

Ziel dieser Studie war es, den Status von Wissenserwerb, Wissensstand und Wissenstransfer innerhalb und zwischen den Berufsgruppen, die phlebologische Kompressionsverbände (PKV) anwenden, in Deutschland zu erfassen. Darauf aufbauend, sollte ermittelt werden, welche Möglichkeiten und Hürden diesbezüglich im Themenbereich der phlebologischen Kompressionstherapie in Deutschland bestehen.

## Material und Methoden

### Teilnehmer der Studie

Diese Querschnittsstudie wurde von Oktober 2022 bis März 2023 deutschlandweit durchgeführt. Die Befragung wandte sich an Berufsgruppen, die PKV anwenden. Sie erfolgte auf persönliche Ansprache und im Rahmen von Wundexpertenkursen, Workshops und Vorträgen auf Fachtagungen und Kongressen sowie hausinternen Mitarbeiterschulungen im Themenbereich Wundversorgung und Kompressionstherapie.

### Einschlusskriterien

Einschlusskriterien waren die Zugehörigkeit zu einer phlebologisch tätigen Berufsgruppe und Volljährigkeit. Mit der Rückgabe des ausgefüllten Fragebogens erklärten sich die Teilnehmer mit der Teilnahme an der Studie einverstanden.

### Fragebogen

Die Teilnehmer erhielten einen papierbasierten Fragebogen. Im ersten Teil wurden allgemeine Angaben zu Charakteristiken der Teilnehmer abgefragt. In 3 weiteren Sektionen wurden dann die Themenbereiche Wissenserwerb, Wissensstand und Wissenstransfer im Themengebiet Kompressionstherapie erhoben. Alle Angaben wurden von den Teilnehmern selbstberichtet. Zur Prüfung der Praktikabilität wurde der Fragebogen vorab einem Pre-Test unterzogen, an dem 8 Testkandidaten teilnahmen.

### Datenmanagement und Auswertungen

Die erhobenen Daten wurden zunächst in eine Tabelle in Windows Excel eingetragen und anschließend mit SPSS v. 27.0 für Windows (IBM, Armonk, NY, USA) ausgewertet. Bei der Eintragung wurden Antworten auf Plausibilität geprüft. Die Auswertung erfolgte deskriptiv. Je nach Datenniveau wurden Häufigkeiten und Prozente bzw. Mittelwert, Standardabweichung, Median und Spannweite berechnet. Hinsichtlich der Fragen zu Fachzeitschriften und Fachbüchern sowie zur Kenntnis über AWMF(Arbeitsgemeinschaft der Wissenschaftlichen Medizinischen Fachgesellschaften e. V.)-Leitlinien und DNQP(Deutsches Netzwerk für Qualitätsentwicklung in der Pflege)-Expertenstandards, in denen die Kompressionstherapie thematisiert wird, sowie Datenbanken wurde zusätzlich eine Subgruppenanalyse zwischen Medizinern (Ärzte) und Nicht-Medizinern (Pflegefachkräfte, medizinische Fachangestellte, Sonstige) durchgeführt. Dafür wurden alle Fragen dichotomisiert (ja vs. nein). Aufgrund der unterschiedlichen Subgruppengrößen wurden nur Häufigkeit und Prozent berechnet und keine Signifikanzprüfungen durchgeführt.

## Ergebnisse

Insgesamt 522 Teilnehmer, die über alle Bundesländer verteilt waren, füllten den Fragebogen aus (Tab. 1). Das Alter betrug im Durchschnitt 42,9 Jahre (Standardabweichung [SD] 11,31; Median 42; Range 18–71). Sie hatten im Mittel 19,3 Jahre Berufserfahrung (SD 11,43; Median 18,5; Range 0–47) und erstellten wöchentlich durchschnittlich 6,7 Kompressionsversorgungen (SD 9,01; Median 3; Range 0–70). Insgesamt 483 (92,5 %) Teilnehmer waren Nicht-Mediziner und 38 (7,5 %) Teilnehmer waren Mediziner.

**Tab. 1:** Teilnehmermerkmale

Variable (*n* = 522)	Antwortkategorie	n	%
Geschlecht	Männlich	91	17,4
	Weiblich	428	82,0
	Divers	3	0,6
Zuletzt erworbener Berufsabschluss	Gesundheits- und Krankenpfleger/in bzw. Pflegefachfrau/-mann bzw. Krankenschwester	274	52,5
	Altenpfleger/in	132	25,3
	Kindergesundheits- und Krankenpfleger/in	6	1,1
	Pflegestudium	14	2,7
	Medizinische/r Fachangestellte/r	55	10,5
	Arzt/Ärztin^a^	39	7,5
	Sonstige^b^	2	0,4
Tätigkeitsbereich (Mehrfachantworten möglich)	Klinik	212	40,6
	Arztpraxis	77	14,8
	Wundzentrum	18	3,4
	Ambulante Pflege	125	23,9
	Stationäre Altenpflege	65	12,5
	Homecare/Sanitätshaus	15	2,69
	Sonstige^c^	19	3,6
Weiterqualifizierung (Mehrfachantworten möglich)	Wundexperte/in ICW	396	75,9
	Fachtherapeut/in Wunde ICW	28	5,4
	Pflegetherapeut/in Wunde ICW	11	2,1
	Wundexperte/in DEKRA	2	0,4
	Wundassisstent/in WAcert DGfW	0	0,0
	Wundtherapeut/in WTcert DGfW	0	0,0
	Wundmanager/in nach Kammerlander	1	0,2
	Pflegeexperte/in Stoma, Kontinenz und Wunde FgSKW	4	0,8
	Sonstige^d^	6	1,2

*ICW* Initiative Chronische Wunden e. V., *DEKRA* Deutscher Kraftfahrzeug-Überwachungs-Verein, *WAcert* Zertifizierter Wundassistent/WAcert DGfW, *WTcert* Zertifizierter Wundtherapeut/WTcert DGfW, *DGfW* Deutsche Gesellschaft für Wundheilung und Wundbehandlung e. V., *FgSKW* Fachgesellschaft Stoma, Kontinenz und Wunde

^a^Freitextangaben: Allgemeinmedizin, Angiologie, Chirurgie, Dermatologie, Diabetologie, Phlebologie, Hausarzt, Gefäßchirurgie, Innere/Geriatrie, Neurologie

^b^Freitextangaben: Podologe/in, Auszubildende/r zur Pflegefachfrau/zum Pflegefachmann

^c^Freitextangaben: Ausbildung Pflege, medizinisches Versorgungszentrum (MVZ), außerklinische Intensivpflege, Dialysezentrum, Hospiz, gesetzliche Krankenversicherung, Psychiatrie, Wundauflagenhersteller, Podologie-Praxis, Schulungswesen, spezialisierte ambulante Palliativpflege (SAPV), Wundambulanz

^d^Freitextangaben: Ärztliche/r Wundexperte/in ICW, European Fellow for Wound Healing (EAFWH), Geprüfter Wundberater/in AWM Akademie für Wundmanagement, Wundmanager/in TÜV Rheinland, Pflegeexperte/in chronische Wunden

### Wissenserwerb in der Kompressionstherapie

Fachzeitschriften, die Kompressionstherapie thematisieren, wurden von 55,7 % der Teilnehmer gelesen. Hiervon lasen 29,4 % bis zu 2‑mal/Jahr und 26,3 % bis zu 6‑mal/Jahr oder häufiger Fachzeitschriften. Von den Teilnehmern hatten 19,2 % eine, 5,0 % zwei, 1,1 % drei und 0,6 % mehr als drei Fachzeitschriften abonniert. Fachbücher, die Kompressionstherapie thematisieren, besaßen 37,0 %, wovon der Großteil (60,1 %) angab, dass die meisten davon unter 5 Jahre alt sind. Der Anteil an Teilnehmern, die Fachzeitschriften lasen (71,8 % vs. 54,2 %), Fachzeitschriften abonniert hatten (53,8 % vs. 23,6 %) und Fachbücher besaßen (56,4 % vs. 35,4 %), war unter Medizinern höher als unter Nicht-Medizinern.

An internen Fortbildungen, die unter anderem das Thema Kompressionstherapie behandeln, nahmen 41,2 % mindestens jährlich, 9,0 % alle 2 Jahre, 5,9 % deutlich seltener und 43,9 % gar nicht teil. Zu 67,6 % hatten die internen Fortbildungen einen Umfang von 1–2 h, zu 23,9 % von einem halben Tag und zu 8,5 % von bis zu einem Tag und länger. Die Inhalte (Mehrfachnennungen möglich) wurden zu 52,6 % von Kollegen aus der Einrichtung, zu 45,7 % von externen Dozenten und zu 56,0 % herstellerseitig im Rahmen von Produktschulungen oder durch Mitarbeiter von Sanitätshäusern oder Homecare-Unternehmen vermittelt.

An externen Fortbildungen, die unter anderem das Thema Kompressionstherapie behandeln, nahmen 72,0 % mindestens jährlich, 10,9 % alle 2 Jahre, 5,6 % deutlich seltener und 11,1 % gar nicht teil. Zu 71,5 % hatten die externen Fortbildungen einen Umfang von einem Tag oder länger und zu 20,5 % von bis zu einem halben Tag. Die Inhalte (Mehrfachnennungen möglich) wurden zu 92,9 % von externen Dozenten, zu 30,3 % herstellerseitig im Rahmen von Produktschulungen oder durch Mitarbeiter von Sanitätshäusern bzw. Homecare-Unternehmen und zu 10 % über Vereine und Fachgesellschaften vermittelt.

An Online-Fortbildungen, die unter anderem das Thema Kompressionstherapie behandeln, nahmen, 19,2 % mindestens jährlich, 10 % alle 2 Jahre, 4,3 % deutlich seltener teil und 66,3 % gar nicht. Die Online-Fortbildungen hatten zu 88,0 % einen Umfang von bis zu einem halben und zu 11,5 % von einem Tag. Diese waren meist herstellerneutrale E‑Learnings (61,5 %) oder Web-Seminare (53,4 %), teilweise aber auch herstellerinitiierte E‑Learnings (19,0 %) oder Web-Seminare (25,9 %).

**Abb. 1:**
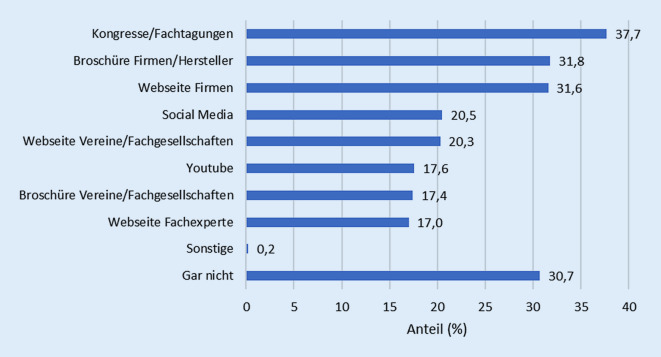
Informationsquellen zum Wissenserwerb (*n* = 522, Mehrfachnennungen möglich, % Anteil)

Etwa ein Drittel (30,7 %) gab an, keine Informationsquellen zum Wissenserwerb zu nutzen. Als häufigste Informationsquellen (Mehrfachnennungen möglich) wurden Kongresse und Fachtagungen (37,7 %), Websites (31,6 %) sowie Broschüren von Firmen bzw. Herstellern (31,8 %) genannt (Abb. 1).

### Wissensstand in der Kompressionstherapie

Die Teilnehmer gaben an, dass das Thema Kompressionstherapie in ihrer Ausbildung bzw. ihrem Studium zu 43,3 % gar nicht, zu 35,1 % in 1 bis 2 Unterrichtsstunden, zu 13,4 % in 3 bis 4 Unterrichtsstunden und zu 8,3 % in einem Zeitraum von einem Tag oder länger vermittelt wurde. Die relevanten Leitlinien der AWMF (Kompressionstherapie, Ulcus cruris venosum, Lymphödem und intermittierende pneumatische Kompression) kannten 6,7 % und konnten diese benennen. Den DNQP-Expertenstandard Pflege von Menschen mit chronischen Wunden, der auf das Themengebiet phlebologische Kompressionstherapie eingeht, kannten und benannten 16,3 %. Themenrelevante Datenbanken wurden von 7,6 % angegeben. Hierzu gehörten PubMed/MEDLINE, Google Scholar, Cochrane, Embase und Cinahl. Während anteilig deutlich mehr Mediziner als Nicht-Mediziner die AWMF-Leitlinien (43,6 % vs. 3,7 %) und Datenbanken kannten (38,5 % vs. 4,8 %), war der DNQP-Expertenstandard mehr Nicht-Medizinern als Medizinern bekannt (17,4 % vs. 2,6 %).

Bei den Angaben der Teilnehmer, ob sie Fachgesellschaften und Vereine kennen, die sich mit dem Thema phlebologische Kompressionstherapie beschäftigen oder sich in ihnen engagieren, wurden am meisten die Initiative Chronische Wunden (ICW) e. V. und das Wundzentrum Hamburg e. V. benannt (Tab. 2).

**Tab. 2:** Kenntnis, Mitgliedschaft und Engagement in Institutionen

Kennen Sie die folgenden Institutionen? (*n* = 522)	Bekannt		Mitglied		Engagement in Arbeitsgruppen	
	*n*	%	*n*	%	*n*	%
Initiative Chronische Wunden (ICW)	457	87,5	95	18,2	25	4,8
Deutsche Gesellschaft für Wundheilung und Wundbehandlung (DGfW)	235	45,0	2	0,4	0	0,0
Fachgesellschaft Stoma, Kontinenz und Wunde (FgSKW)	144	27,6	4	0,8	4	0,8
Wundzentrum Hamburg	334	64,0	36	6,9	5	1,0
Deutsche Gesellschaft für Phlebologie^a^	110	21,1	8	1,5	2	0,4
Deutsche Gesellschaft für Lymphologie^a^	87	16,7	3	0,6	0	0,0
Austrian Wound Association (AWA)	61	11,7	0	0,0	1	0,2
Schweizerische Gesellschaft für Wundbehandlung (SAfW)	56	10,7	0	0,0	2	0,4
WundDACH	137	26,2	2	0,4	1	0,2
European Wound Management Association (EWMA)	119	22,8	3	0,6	2	0,4
Sonstige^b^	4	0,8	–	–	–	–

^a^Die Deutsche Gesellschaft für Phlebologie und die Deutsche Gesellschaft für Lymphologie sind seit dem 01.01.2023 zur Deutschen Gesellschaft für Phlebologie und Lymphologie vereinigt

^b^Freitextangaben: AG Wunde der DDG (Deutsche Diabetes Gesellschaft), Deutsche Gesellschaft für Gefäßmedizin und Gefäßchirurgie, European Association of Fellows Woundhealing, Deutscher Wundrat, International Compression Club

Auf die Frage, welche Versorgungsoptionen die Teilnehmer anwenden (Tab. 3), berichteten die meisten, dass sie oft oder regelmäßig medizinische Kompressionsstrümpfe (81,6 %) nutzen, gefolgt von Kurzzugbinden mit Unterpolsterung (53,3 %) und Kurzzugbinden ohne Unterpolsterung (37,4 %). In der Eigeneinschätzung ihrer Kompetenz hinsichtlich der phlebologischen Kompressionstherapie bewerteten sich 38,7 % als Anfänger, 49,0 % als Fortgeschrittene und 12,3 % als Experten.

**Tab. 3:** Nutzung von Versorgungsoptionen in der Kompressionstherapie

Anwendung im Versorgungsalltag (*n* = 522)	Gar nicht		Selten		Oft		Regelmäßig	
	*n*	%	*n*	%	*n*	%	*n*	%
Kurzzugbinden ohne Unterpolsterung	190	36,4	134	25,7	99	19,0	96	18,4
Kurzzugbinden mit Unterpolsterung	86	16,5	158	30,3	110	21,1	168	32,2
Mehrkomponentensysteme	258	49,4	131	25,1	74	14,2	55	10,5
Medizinische adaptive Kompressionssysteme	364	69,7	104	19,9	30	5,7	20	3,8
Ulkus-Strumpfsysteme	237	45,4	147	28,2	85	16,3	51	9,8
Medizinische Kompressionsstrümpfe	34	6,5	62	11,9	137	26,2	289	55,4

### Wissenstransfer in der phlebologischen Kompressionstherapie

Es gaben 31,2 % an, dass ihre Einrichtung keinen Standard zur phlebologischen Kompressionstherapie implementiert hat, weiteren 23,6 % war dies nicht bekannt. Die Weitergabe ihres eigenen Wissens erfolgte nach Angaben der Teilnehmer zu 67,2 % im Kollegenkreis und zu 28,9 % gar nicht. Weiterer Wissenstransfer fand über Dozententätigkeit (7,5 %), Publikationen (2,7 %) sowie im Internet (3,0 %) statt.

**Abb. 2:**
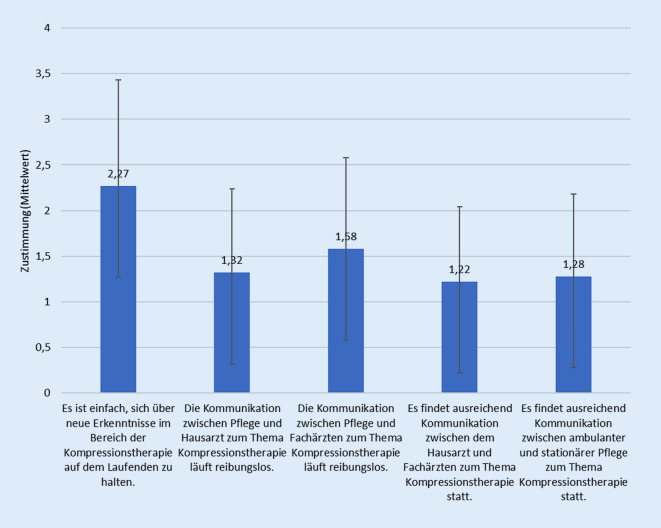
Zustimmung zu Aussagen über Wissenstransfer in der Kompressionstherapie. Skala von 0 (stimme nicht zu) bis 4 (stimme zu)

Auf einer Skala von 0 (stimme nicht zu) bis 4 (stimme zu) schätzten die Teilnehmer ein, wie einfach es ist, sich auf dem Laufenden zum Thema phlebologische Kompressionstherapie zu halten, und wie gut die Kommunikation zwischen unterschiedlichen Akteuren funktioniert (Abb. 2).

Zudem konnten die Teilnehmer am Ende des Fragebogens noch Einschätzungen zum Wissenstransfer per Freitext eintragen, wovon 5 Personen Gebrauch machten. Sie kritisierten fehlendes Fachwissen bei allen Akteuren, insbesondere bei der Vermittlung durch Lehrer für Pflegeberufe und die kaum vorhandene aktuelle Fachliteratur. Zudem wurde der Wunsch nach vertieftem Wissen und einer Weiterbildung im Themenbereich Kompressionstherapie geäußert.

## Diskussion

Das Wissen der Versorger in der phlebologischen Kompressionstherapie ist seit Jahren ungenügend, aktuelles Material ist häufig nicht bekannt, und eine sach- und fachgerechte Umsetzung erfolgt selten [7–12]. Bisherige Studien fokussieren oft auf die Verbesserung der Ergebnisse einer phlebologischen Kompressionstherapie durch Änderung und Anpassung der Materialien [13–15]. Ein nicht einsetzender Therapieerfolg liegt aber nicht nur an den verwendeten Versorgungsoptionen, sondern auch daran, wie diese von den Anwendern eingesetzt werden [16]. Ob der Einsatz sachgerecht erfolgt, hängt von dem Wissen und von den praktischen Fertigkeiten der Anwender ab. Hierbei ist entscheidend, ob das individuelle Wissen auch aktuell ist und regelmäßig ergänzt wurde. Daher werden in dieser Studie erstmalig Wissenserwerb, Wissensstand und Wissenstransfer bei Versorgern in Deutschland im Themenbereich der phlebologischen Kompressionstherapie unter verschiedenen Aspekten erhoben. Aus der gemeinsamen Betrachtung dieser 3 Aspekte ergeben sich Ansätze dafür, wie in den Gesundheitsberufen der Wissenstransfer von der Wissenschaft in die Praxis erfolgen könnte. Unter dem Begriff Wissenstransfer betrachten wir in dieser Studie allerdings in erster Linie die Weitergabe von Wissen innerhalb der Gruppe der Versorger.

### Wissenserwerb

Mangelhafter Wissensstand resultiert aus unzureichendem oder unsachgemäßem Wissenserwerb. Hierbei können ungenügende Strukturen zusätzlich hindernd wirken, aber zunächst ist Wissenserwerb eine individuelle Leistung. Fachzeitschriften sind eine naheliegende Wissensquelle für Gesundheitsberufe. Allerdings bezog nur jeder Vierte mindestens eine Fachzeitschrift über ein Abonnement. Insgesamt lasen nur 16,1 % der Teilnehmer 6‑mal/Jahr oder häufiger eine Fachzeitschrift, die unter anderem über das Thema Kompressionstherapie berichtete. Fachbücher beleuchten einen Themenbereich intensiver als Zeitschriften, sind aber meist nicht so aktuell. Knapp zwei Drittel der Teilnehmer hatten keine Fachbücher, die unter anderem phlebologische Kompressionstherapie thematisieren. Ein Großteil der befragten Versorger hat also keinen oder nicht ausreichenden Zugang zu aktueller Literatur in diesem Fachgebiet.

Um ein Mindestmaß an aktuellem Fachwissen zu gewährleisten, ist die regelmäßige Teilnahme an Fortbildungen für Ärzte nach § 95d Sozialgesetzbuch (SGB) V und für Pflegkräfte beispielsweise nach Pflegeberufegesetz (PflBG) verpflichtend. Entsprechend besuchten auch viele der Befragten mindestens 1‑mal pro Jahr eine Fortbildung, die unter anderem phlebologische Kompressionstherapie thematisierte. Davon wurde allerdings mindestens jede Dritte von Hersteller- bzw. Händlerseite ausgerichtet. Diese Akteure sind meist nicht frei von Interessenkonflikten. Zudem unterliegen diese Veranstaltungen nicht der Kontrolle von Fachgesellschaften, beispielsweise durch vorgegebene leitliniengerechte Inhalte und Zertifizierung. Vergleichbar mit unseren Resultaten konnte in einer britischen Studie mit 323 Medizinstudenten aus 18 britischen Universitäten gezeigt werden, dass auch das wichtige Thema der Wundbehandlung im Medizinstudium kaum (68,4 %) oder gar nicht (32,6 %) unterrichtet wurde. Im Durchschnitt erhielten die Studenten 2,25 h strukturierten, vorklinischen Unterricht und insgesamt 1,0 h klinischen Unterricht [22]. Diese Studie belegt zudem das deutliche Defizit bei der real durchgeführten Wundausbildung im Vergleich zu den Erwartungen der Studenten.

Bei sonstigen Informationsquellen wurden zu knapp einem Drittel Angebote von Herstellern genutzt, z. B. Firmenbroschüren und -websites. Da solche Medien oft visuell ansprechender aufbereitet und textlich verständlicher formuliert sind, ist anzunehmen, dass sie deshalb mehr konsumiert werden als vergleichbare wissenschaftliche Angebote z. B. von Vereinen, Fachgesellschaften und sonstigen gemeinnützigen Organisationen.

### Wissensstand

Obwohl die Therapie durch den Einsatz von Leitlinien optimiert werden kann, werden diese in der Praxis oft nicht befolgt. Die meistgenannten Gründe hierfür sind keine oder mangelnde Kenntnis oder fehlende Akzeptanz der Leitlinien [17]. Unsere Ergebnisse bestätigen diese Daten. Die themenbezogenen AWMF-Leitlinien und der DNQP-Expertenstandard Pflege von Menschen mit chronischen Wunden waren kaum bekannt. Allerdings gab es berufsgruppenspezifische Unterschiede. So waren die Mediziner erheblich vertrauter mit den themenbezogenen AWMF-Leitlinien und kannten auch deutlich häufiger Datenbanken. Gründe hierfür können sein, dass Mediziner bereits im Studium mit dem Gebrauch von Datenbanken vertraut gemacht werden und die AWMF-Leitlinien für ihre Berufsausübung eine wesentliche Grundlage sind. Dies könnte analog erklären, warum der DNQP-Expertenstandard unter Nicht-Medizinern deutlich bekannter war.

Oft verlassen sich Praktiker bevorzugt auf ihr bestehendes Wissen und ihre eigenen Erfahrungen [18]. Allerdings ist auch die Vorbildung hinsichtlich der phlebologischen Kompressionstherapie in der Breite fraglich. Sie wird in der universitären medizinischen Ausbildung weitestgehend nicht und in der pflegerischen Ausbildung im Durchschnitt in 1 bis 2 Unterrichtsstunden gelehrt [19]. Diese Daten wurden in der vorliegenden Studie bestätigt. Knapp ein Fünftel der Teilnehmer gab zwar an, mehr Unterricht zur Kompressionstherapie in der Ausbildung gehabt zu haben, allerdings erinnerten sich 43,3 % an gar keinen Unterricht zu diesem Thema. Dennoch schätzten sich 61,3 % als Fortgeschrittene und Experten ein. Die im Abschnitt „Wissensstand in der Kompressionstherapie“ beschriebenen Daten zu verwendeten Therapieoptionen zeigen, dass diese Selbsteinschätzung größtenteils nicht zutrifft. So wurden als Materialien für die Erstellung von PKV größtenteils Kurzzugbinden mit und ohne Unterpolsterung oft und regelmäßig verwendet. Die laut AWMF-Leitlinie anwenderfreundlicheren, effizienteren und durch Patienten besser akzeptierten Mehrkomponentensysteme und medizinische adaptive Kompressionssysteme [20] wurden hingegen erheblich seltener oft und regelmäßig genutzt. Datenbanken mit wissenschaftlichen Erkenntnissen zum Themenbereich der phlebologischen Kompressionstherapie waren nur 7,6 % der Befragten bekannt. Allerdings bestehen bei deren Nutzung zum Teil erhebliche Barrieren hinsichtlich eines freien Zugangs, der Sprache und der Verständlichkeit der verfügbaren Einträge. Demgegenüber sind AWMF-Leitlinien und DNQP-Expertenstandards vergleichsweise einfach und in deutscher Sprache verfügbar. Obwohl ca. 80 % der Teilnehmer mindestens eine von einer Fachgesellschaft zertifizierte Weiterqualifizierung in der Versorgung von Menschen mit chronischen Wunden, die auch phlebologische Kompressionstherapie thematisiert, absolviert hatten, waren solche grundlegenden Kenntnisse, die Bestandteil der jeweiligen Curricula sind, nicht ausreichend vorhanden.

### Wissenstransfer

Die konsequente Beachtung von einrichtungsinternen evidenzbasierten Standards kann beispielsweise die Abheilung von Ulcus cruris venosum (UCV) beschleunigen und das Auftreten von Rezidiven mindern [21]. In dieser Studie gaben mehr als die Hälfte der Teilnehmer an, dass in ihrer Einrichtung entweder kein Standard zur Kompressionstherapie vorliegt oder ihnen ein solcher nicht bekannt war. Somit waren die meisten der Befragten also in einer Einrichtung tätig, die keinen Standard oder einen solchen nicht ausreichend implementiert hatte. Gut zwei Drittel der Teilnehmer gaben an, ihr Wissen zur phlebologischen Kompressionstherapie im Kollegenkreis weiterzugeben. Zur interdisziplinären und interprofessionellen Kommunikation sowie zum Austausch zwischen den Bereichen ambulant/stationär gab es im Wesentlichen negative Einschätzungen. Dies ist alarmierend, denn unzureichende Kommunikation zwischen den Akteuren kann zu Therapiebrüchen führen und somit erhebliche Auswirkungen auf den Therapieverlauf und in der Folge auf die Lebensqualität und die Adhärenz der Patienten haben sowie die Kosten für die Versorgung steigern [21]. Kommunikative Defizite entsprechend zu identifizieren und auszugleichen bedeutet für alle Beteiligten zusätzlichen zeitlichen Aufwand, führt zu Unzufriedenheit und Stress.

### Limitationen

Es wurde zwar eine große Anzahl an Teilnehmern rekrutiert, allerdings bilden die dargestellten Daten nicht den tatsächlichen Wissensstand und alle Arten des Wissenstransfers in der phlebologischen Kompressionstherapie ab. Es entsteht eine Verzerrung, da die Daten zum einen in Kursen bzw. bei Kongressen mit dem Fokus Wund- und Kompressionsversorgung gesammelt wurden und zum anderen von den Teilnehmern selbstberichtet wurden. Es ist davon auszugehen, dass diese Stichprobe gehäuft ein grundlegendes Interesse an der phlebologischen Kompressionstherapie hat. Ablesbar sind somit der Wissensstand und die Wege des Wissenstransfers aus Sicht von bereits spezialisierten bzw. qualifizierten und engagierten Versorgern. Trotz dieser Verzerrungen zeigen die Ergebnisse selbst in dieser Gruppe erhebliche Defizite im Hinblick auf den Wissensstand. Das Wissen zum Themenkomplex Kompressionstherapie ist in der Breite demnach als noch deutlich schlechter einzuschätzen.

## Fazit

Die Ergebnisse zeigen, dass auch weiterqualifizierte Mediziner und Nicht-Mediziner relevante Leitlinien und Expertenstandards kaum kennen, insbesondere solche, die vermeintlich der anderen Berufsgruppe zugeordnet sind. Für eine sachgerechte Versorgung auf dem aktuellen Stand der Wissenschaft und ein gemeinsames koordiniertes Vorgehen auf Augenhöhe im Themenbereich Kompressionstherapie wäre es förderlich, wenn Nicht-Mediziner auch mit AWMF-Leitlinien und Mediziner mit den DNQP-Expertenstandards vertraut sind. Auch weitere Quellen des Wissenserwerbs wurden insgesamt nur im geringen Umfang genutzt. Verschiedene Studien belegen, dass den Versorgern aktuelle Therapieoptionen der phlebologischen Kompressionstherapie nicht bekannt sind. Zudem werden die Materialien, die ihnen vertraut sind, insbesondere Kurzzugbinden, nicht sachgerecht angelegt [5, 10, 19]. Die Ergebnisse unserer Studie können dazu beitragen, mögliche Gründe für die oft beobachtete Kluft zwischen Wissenschaft und Praxis zu identifizieren und Lösungswege aufzuzeigen. Bereits in der Ausbildung könnte beispielsweise vermittelt werden, dass die Lektüre aktueller fachbezogener Publikationen Bestandteil des Berufslebens ist. Auch sollte thematisiert werden, welche Quellen und Fortbildungsangebote sinnvoll sind und dass der Austausch über berufliche Sachverhalte unter den Kollegen zum Praxisalltag gehört. Durch eigenständigen Wissenserwerb nach dem Prinzip des lebenslangen Lernens und die barrierefreie interdisziplinäre und interprofessionelle Kommunikation lässt sich der Wissenstransfer in den Gesundheitsberufen optimieren.
